# Power analysis dataset for QCA based multiplexer circuits

**DOI:** 10.1016/j.dib.2017.03.001

**Published:** 2017-03-09

**Authors:** Md. Abdullah-Al-Shafi, Ali Newaz Bahar, Peer Zahoor Ahmad, Firdous Ahmad, Mohammad Maksudur Rahman Bhuiyan, Kawsar Ahmed

**Affiliations:** a Institute of Information Technology (IIT), University of Dhaka, Bangladesh; b Department of Information and Communication Technology, Mawlana Bhashani Science and Technology University, Bangladesh; c Department of Computer Science, University of Kashmir, 190006 J&K, India; d Department of Electronics & IT, University of Kashmir, 190006 J&K, India; e University Grants Commission of Bangladesh, Bangladesh

**Keywords:** Quantum-dot cellular automata, Multiplexer, Power dissipation, QCAPro

## Abstract

Power consumption in irreversible QCA logic circuits is a vital and a major issue; however in the practical cases, this focus is mostly omitted.The complete power depletion dataset of different QCA multiplexers have been worked out in this paper. At −271.15 °C temperature, the depletion is evaluated under three separate tunneling energy levels. All the circuits are designed with QCADesigner, a broadly used simulation engine and QCAPro tool has been applied for estimating the power dissipation.

**Specifications Table**

**Table:** 

Subject area	*Electronics*
More specific subject area	*Nano-electronics*
Type of data	*Table, figure*
How data was acquired	*QCADesigner and Hamming distance process have been applied to attain the data set*
Data format	*Analyzed*
Data accessibility	*Data is within this article*

**Value of the data**

• Computer memories, communication systems and other circuit structures can be utilized for computational analysis using this study in terms of power consumption.
• The presented data analysis can support the researchers to examine the energy analysis of complex network systems.
• It can be utilized to estimate polarization error and non adiabatic switching power loss in QCA reversible designs.

## 1 Data

**Fig. 1:**
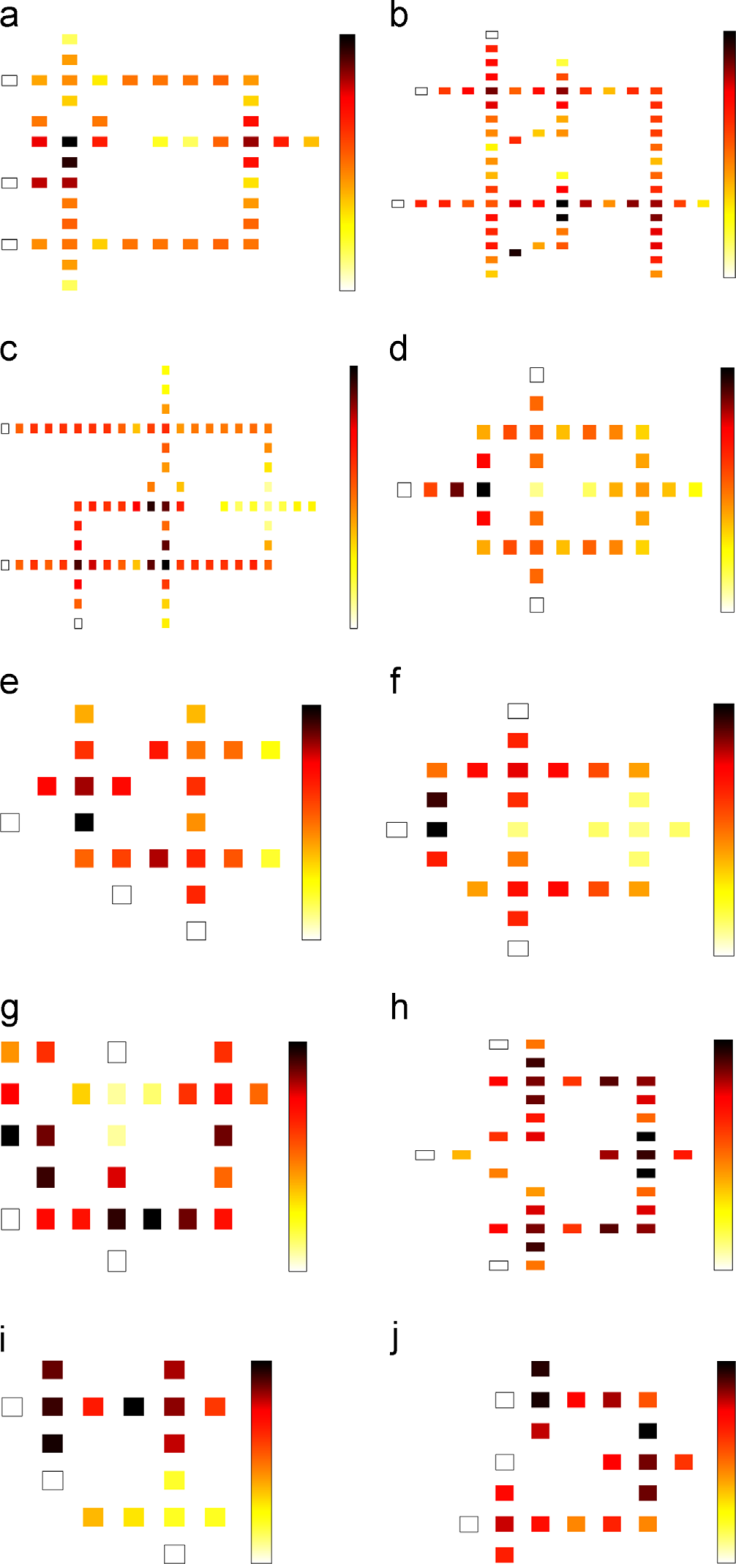
The power dissipation maps of multiplexer in (a) Ref. [1] (b) Ref. [2] (c) Ref. [3] (d) Ref. [4] (e) Ref. [5] (f) Ref. [6] (g) Ref. [7] (h) Ref. [8] (i) Ref. [9] and (j) Ref. [10] at 2 K temperature with 0.5 Ek tunneling energy level.

In this paper, power dissipation analysis of different multiplexer circuits presented in [1], [2], [3], [4], [5], [6], [7], [8], [9], [10], have been investigated in Table 1 at three different tunneling energy levels like $\gamma =0.5E_{k},\gamma =1.0E_{k}$ and $\gamma =1.5E_{k}.$ The energy dissipation map which includes leakage power dissipation, switching power dissipation and average power dissipation of various QCA multiplexers have been shown in Fig. 1.

## 2 Experimental design, materials and methods

### 2.1 Analysis of power dissipation

For estimating the power dissipation of reported multiplexers [1], [2], [3], [4], [5], [6], [7], [8], [9], [10] QCAPro; a power analyzing tools for QCA design has been applied. This tool estimate polarization error and non-adiabatic switching power loss in Quantum-dot Cellular Automata (QCA) circuits. It uses a fast approximation based technique to estimate highly erroneous cells in QCA circuit design. In our study, power estimation of all the multiplexers has been achieved at a stable temperature *T*= −271.15 °C. The power dissipation by a QCA cell is calculated using the Hartree–Fock mean-field approach approximation which is illustrated as [11], [12], [13], [14], [15]

(1) $$H=\left[\begin{array}{cc} \frac{{-E}_{k}}{2}\sum_{i}C_{i}f_{i,j} & -\gamma \\ -\gamma & \frac{E_{k}}{2}\sum_{i}C_{i}f_{i,j} \end{array}\right]=\left[\begin{array}{cc} \frac{{-E}_{k}}{2}\left(C_{j-1}+C_{j+1}\right) & -\gamma \\ -\gamma & \frac{E_{k}}{2}\left(C_{j-1}+C_{j+1}\right) \end{array}\right]$$

According to the upper bound power dissipation model [14] the power dissipation by a QCA cell is given as

(2) $$P_{\mathrm{diss}}=\frac{E_{\mathrm{diss}}}{T_{\mathrm{cc}}}\langle \frac{\hbar }{{2T}_{\mathrm{cc}}}{\vec{Г}}_{+}\times \left[-\frac{{\vec{Г}}_{+}}{\left|{\vec{Г}}_{+}\right|}\tan h\left(\frac{\hbar \left|{\vec{Г}}_{+}\right|}{k_{B}T}\right)+\frac{{\vec{Г}}_{-}}{\left|{\vec{Г}}_{-}\right|}\tan h\left(\frac{\hbar \left|{\vec{Г}}_{-}\right|}{k_{B}T}\right)\right]\rangle$$

## Figures and Tables

**Table 1:** Energy dissipation analysis of multiplexers at three different tunneling energy levels.

Circuit	Leakage energy dissipation (meV)			Switching energy dissipation (meV)			Total energy dissipation (meV)		
	*0.5 E*_*k*_	*1.0 E*_*k*_	*1.5 E*_*k*_	*0.5 E*_*k*_	*1.0 E*_*k*_	*1.5 E*_*k*_	*0.5 E*_*k*_	*1.0 E*_*k*_	*1.5 E*_*k*_
Multiplexer[1]	12.4	39.16	71.13	66.98	58.57	50.28	79.38	97.73	121.41
Multiplexer [2]	19.35	60.43	108.67	97.45	83.57	70.55	116.8	144	179.22
Multiplexer [3]	20.38	64.9	118.49	122.17	107.15	92.02	142.55	172.05	210.51
Multiplexer[4]	8.53	27.57	50.43	41.63	36.39	31.22	50.16	63.96	81.65
Multiplexer [5]	7.16	20.53	35.68	25.43	21.7	18.29	32.59	42.23	53.97
Multiplexer [6]	6.72	21.58	39.4	26.25	22.93	19.64	32.97	44.51	59.04
Multiplexer [7]	6.79	21.16	38.27	35.22	30.37	25.85	42.01	51.53	64.12
Multiplexer [8]	10.69	31.68	55.42	38.06	31.87	26.49	48.75	63.55	81.91
Multiplexer [9]	4.54	13.88	24.63	11.41	9.77	8.19	15.95	23.65	32.82
Multiplexer [10]	5.5	17.38	31.17	26.83	22.66	18.91	32.33	40.04	50.08
